# Effects of size and position of an unconnected aluminum electrode on bipolar anodization in an AC electric field

**DOI:** 10.1038/s41598-021-01633-4

**Published:** 2021-11-18

**Authors:** Ryo Takeuchi, Hidetaka Asoh

**Affiliations:** Department of Applied Chemistry, Kogakuin University, 2665-1 Nakano, Hachioji, Tokyo, 192-0015 Japan

**Keywords:** Synthesis and processing, Surfaces, interfaces and thin films, Electrochemistry, Surface chemistry

## Abstract

The effects of the size and position of an aluminum bipolar electrode (BPE) on the uniformity of formation of anodic porous alumina in an alternating current electric field were investigated. Anodized specimens were dyed, and the resistance was measured after the specimens were anodized again. Phenomena observed during film formation indicated that the BPEs had unique potential distributions that strongly depended on their length and width. The color variations and electrical resistance of the BPEs were symmetrical and varied from the centers of the BPEs to their ends. When multiple BPEs were processed at the same time, their position in the non-uniform electric field was demonstrated to be an important factor for controlling the uniformity of film formation. The best results were obtained when the BPE was placed at the center of the defined space.

## Introduction

Bipolar electrochemistry deals with reactions occurring on an unconnected conductive object called a bipolar electrode (BPE) placed between outer electrodes in an alternating current (AC) or direct current (DC) electric field (i.e., bipolar cell). It is a unique concept that has attracted wide attention in many research fields ranging from sensing to materials science^1–4^. The experimental setup for bipolar electrochemistry differs from that of a conventional two- or three-electrode electrochemical system:

- The BPE is not directly connected to the power supply;
- The bipolar cell can have an open or closed configuration;
- Electrically coupled faradaic reactions such as reduction and oxidation (i.e., redox) proceed at opposing poles of the BPE; and
- Multiple objects can be treated simultaneously.

Here, we selected a bipolar cell with an open configuration to investigate the effects of the BPE positions on bipolar anodization. In most studies that use an open bipolar cell, the plate-like BPE is horizontally positioned at the center between two driving electrodes^5,6^. Moreover, multiple BPEs in the same external electric field are believed to act the same. Although redox reactions with multiple BPEs have already been reported^7,8^, few studies have focused on the BPE positions between the two driving electrodes. It is natural to put the BPE at the center of the cell, and the influence of the electric field distribution on the redox reactions on the BPE is usually ignored.

Bipolar anodization, which is the formation of metal oxide films such as titanium or aluminum oxide on the BPE, has recently gained intense interest from researchers owing to its potential applications, including sensing and screening applications^9–16^. For practical application as a surface treatment, our group previously formed porous alumina films^17,18^ and a Pt/alumina composite^19^ on aluminum BPEs in both AC and DC electric fields. We also investigated the effective potential gradients for film formation on BPEs in both AC and DC electric fields^20,21^. As a result, the AC field was considered suitable for the formation of a uniform coating in terms of corrosion resistance and decorativeness. In this study, we investigated the effects of the BPE position and size on the uniformity of film formation in an AC field. The results are expected to provide useful insights into the processing of single and multiple BPEs.

## Materials and methods

### Experimental setup

**Figure 1:**
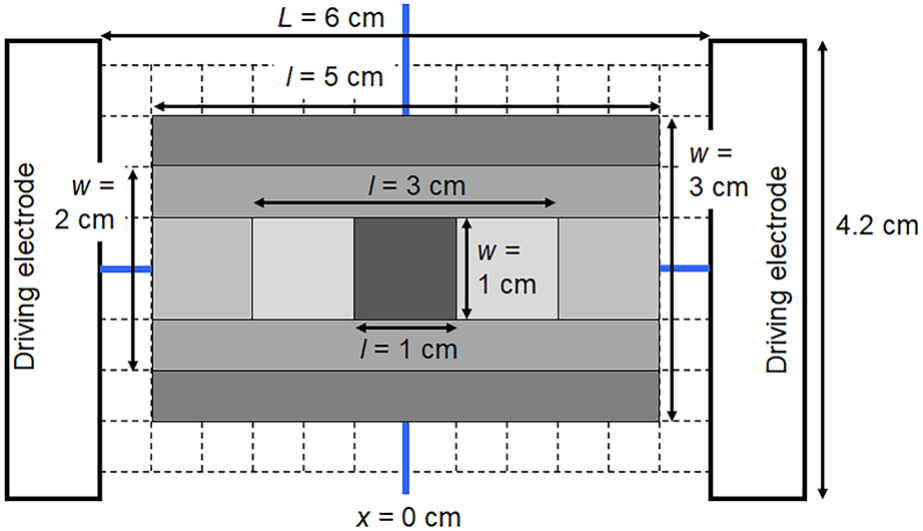
Top-view schematic of the experimental setup for bipolar anodization. *L*, *l*, and *w* denote the distance between the driving electrodes and the length and width of the BPE, respectively. The grid is drawn with a spacing of 5 mm.

High-purity (99.99%) aluminum sheets with a thickness of 400 µm were cleaned by immersion in 5 wt.% NaOH at 60 °C for 20 s and then immersion in 30 vol% HNO_3_ at room temperature for 1 min. Electrolysis in an AC electric field was conducted by using two vertically aligned carbon electrodes with dimensions of 12 cm × 4.2 cm × 0.8 cm and placing an aluminum sheet between them as the BPE. Figure 1 shows a top-view schematic of the electrochemical cell used for AC bipolar anodization. The distance (*L*) between the driving electrodes was 6 cm. The length (*l*) and width (*w*) of each BPE were varied within the ranges of 1–5 cm and 1–3 cm, respectively. The figure also shows all BPEs in order of size. The center of each BPE was aligned with the center of the cell. A detailed description of the cell used to perform AC electrolysis is provided in Fig. S1.

The BPEs were positioned horizontally in the cell for visual assessment of the equipotential surface patterns. One side of each BPE was exposed to the electrolyte, while the other side was covered with Kapton^®^ tape to avoid any reaction. AC electrolysis was performed in 10 mmol dm^−3^ oxalic acid at 20 °C and 60 V for 60 min by using an AA2000XG2 AC power supply (Takasago, Japan). As previously reported, the thickness of the formed films was distinctly affected by frequency. In a range of 10–700 Hz, a thicker oxide film was formed at 150 Hz^17,18^. Therefore, a rectangular waveform with a frequency of 150 Hz was adopted in the present study. The electrolysis conditions were the same as those used in our previous study^20^. For the electrolysis of multiple BPEs, the BPEs were placed on a perforated insulating plate and fixed by double-sided adhesive tape. The effect of the BPE position was then evaluated.

### Characterization

To evaluate the thickness distribution and uniformity of the formed alumina film on each BPE after electrolysis, the aluminum specimens were stained with TAC BLUE-BRL dye (5 g dm^−3^, Okuno Chemical Industries Co., Japan) at 60 °C for 10 min. The thicknesses of the barrier layers in the porous alumina films were electrochemically evaluated by re-anodizing the specimens at 5 A m^−2^ in a solution containing 0.5 mol dm^−3^ boric acid and 0.05 mol dm^−3^ sodium tetraborate at 20 °C. Measurements were performed within 8 mm of the center of each piece, which was cut into a 1 × 1 cm^2^ section, using a sample holder (EC Frontier Co., AE9-1)^20,21^. The voltage–time curves during re-anodization were measured using a digital multimeter equipped with a data acquisition system (Keithley, DMM2700). Changes in the thickness of the barrier layer in each sample were estimated from the voltage jump (*V*_j_), which was obtained by extrapolating the linear region of the voltage–time curve to a re-anodization time of 0^22,23^.

## Results

### AC bipolar anodization of aluminum BPEs with different sizes

The current density–time curves during AC bipolar anodization of the aluminum BPEs were consistent with the results of our previous study^20^. The current density was calculated for the carbon electrode area exposed to the electrolyte, and the exposed surface area of the aluminum BPE was not considered (Fig. 1). Because the carbon driving electrodes were not directly involved in the redox reactions on the BPEs, the current density remained mostly stable regardless of the BPE size.

On carbon driving electrodes, water electrolysis proceeds as follows: oxygen evolves at the anode and hydrogen at the cathode when a sufficiently high voltage is applied to the cell. Since the voltage applied to the driving electrodes was rather high (60 V) compared with the voltage at which water electrolysis occurs, the redox reactions on the carbon electrodes were not the subject of this study. On the BPE, the main film-forming anodic reaction can be expressed as follows:

1 $${\text{2Al }} + {\text{ 3H}}_{{2}} {\text{O }} \to {\text{ Al}}_{{2}} {\text{O}}_{{3}} + {\text{ 6H}}^{ + } + {\text{ 6e}}^{ - }$$

Meanwhile, the main cathodic reaction on the BPE is the following hydrogen evolution reaction:

2 $${\text{2H}}^{ + } + {\text{ 2e}}^{ - } \to {\text{ H}}_{{2}}$$

**Figure 2:**
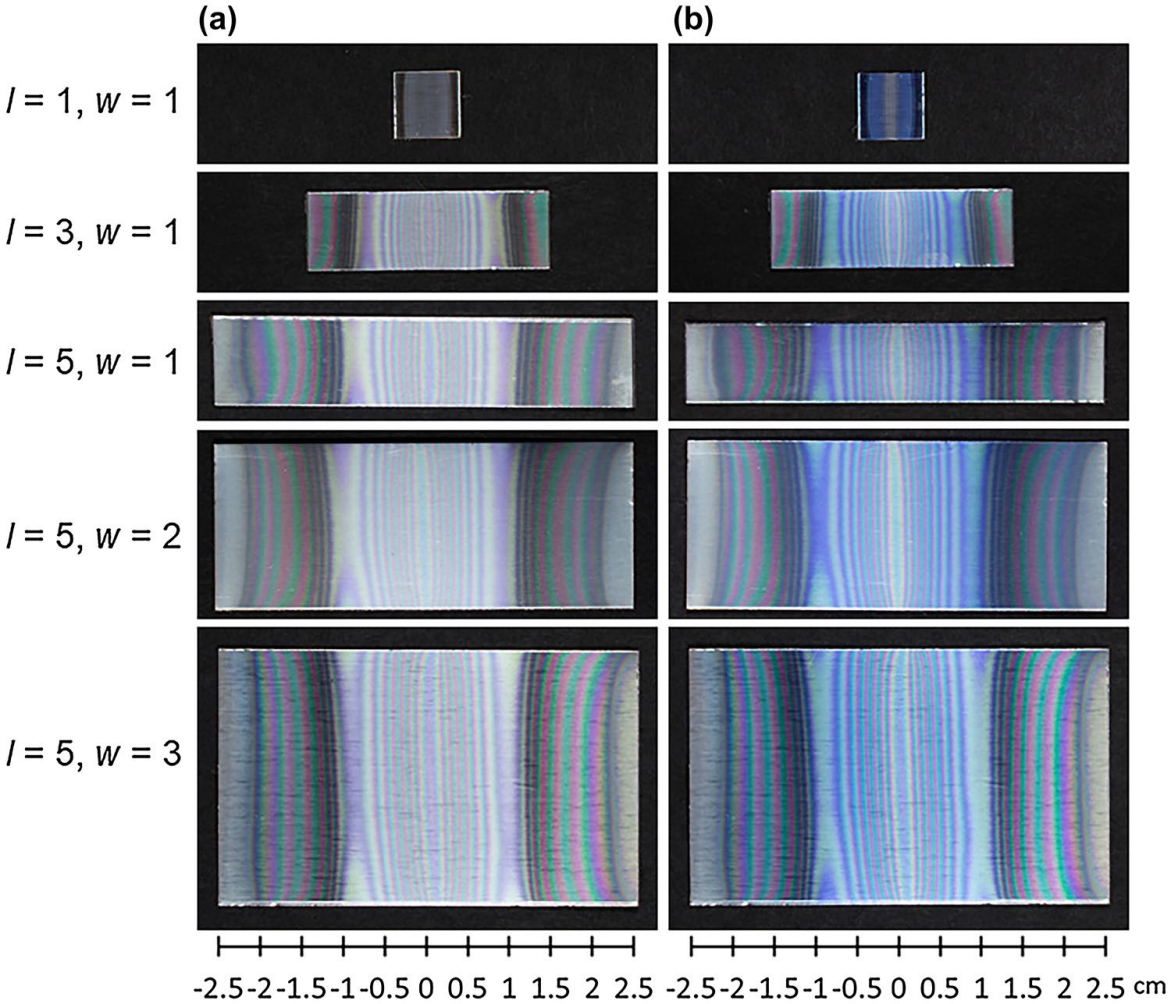
Digital photographs of aluminum sheets **(a)** after AC bipolar anodization in 10 mmol dm^−3^ oxalic acid at 20 °C for 60 min at 60 V and **(b)** after the subsequent dyeing. The length (*l*) and width (*w*) of each BPE were varied in ranges of 1–5 cm and 1–3 cm, respectively.

Figure 2 shows the surface of an anodized specimen before and after dyeing. As the evaluation of the thickness distribution by dyeing was difficult in the case of thin oxide films, the reaction time was set to 60 min to ensure the growth of a sufficiently thick film. Specific interference fringes were observed on all anodized specimens even before they were dyed regardless of the BPE size (Fig. 2a). These interference patterns were also observed in our previous study^20^. The results for a BPE with a length of *l* = 5 cm and width of *w* = 1 cm, which was used as a standard specimen in our previous study^20^, are shown for comparison. When the center of the sample was defined as *x* = 0 cm, a pattern with linear symmetry was observed on each BPE. This result indicates that the thickness of the formed alumina film varied continuously from the center (*x* = 0) to both ends (*x* ~  ± 2.5 cm) of the BPE surface. Some color discrepancies were observed despite the equidistant position from the center. This is probably due to the inaccuracy of the sample placement in the electrochemical cell.

The film thickness distribution can be indirectly estimated by evaluating the color intensity of a dyed specimen because the dye adsorbs to the porous surface, as shown in Fig. 2b. Deeper colors indicate that more dye adsorbed on the porous surface, which would correspond to a thicker film. When BPEs with a variable length of *l* = 1–5 cm and fixed width of *w* = 1 cm were positioned horizontally in the cell, the areas of both ends that retained a metallic luster were different. The short BPEs with *l* = 1 and 3 cm had smaller areas that retained a metallic luster than the BPE with *l* = 5 cm. For the BPE with *l* = 1 cm, both ends were relatively deep blue, and the center was either pale blue or had no blue. Thus, the center of the BPE was concluded to have a relatively thin porous alumina film owing to the low dye adsorption. For the BPE with *l* = 3 cm, although the color 1 cm from the center (*x* ~  ± 1 cm) was more intense than in other areas, both the ends and center were pale blue.

In our previous study, FE-SEM observation was performed to directly examine the detailed film structure^20^. At the edge of the BPE, the film was not a normal anodic porous alumina film with straight pores. Instead, laminate films consisting of closely stacked barrier layers were formed. The films had a minimum thickness of ~ 200 nm compared with other areas as shown in Fig. S2. Because the estimated interfacial potential differences were the highest at the extremities, hydrogen generation suppressed film growth during AC bipolar anodization, resulting in thinner alumina films at the edge of the BPE. Therefore, these areas were not sufficiently stained. The suppression of the anodic reaction (i.e., anodic film formation) based on Eq. (1) as a result of the cathodic reaction according to Eq. (2) has also been observed in conventional AC anodization of aluminum^24^.

For the BPEs with *l* = 5 cm and *w* = 1–3 cm, the color variation along the surface was almost the same regardless of the width. However, the interference fringes showed clear bending. Although the interference fringes were nearly parallel to the surfaces of the driving electrodes, they were not perfectly parallel and distorted at the edges of wide specimens (e.g., *w* = 2 and 3 cm). This may have been caused by the equipotential surface, which is always perpendicular to the electric field lines. If an electric field is uniform, the equipotential surface should be a plane normal to the straight line connecting the driving electrodes.

### Effective potential difference distribution on a BPE

**Figure 3:**
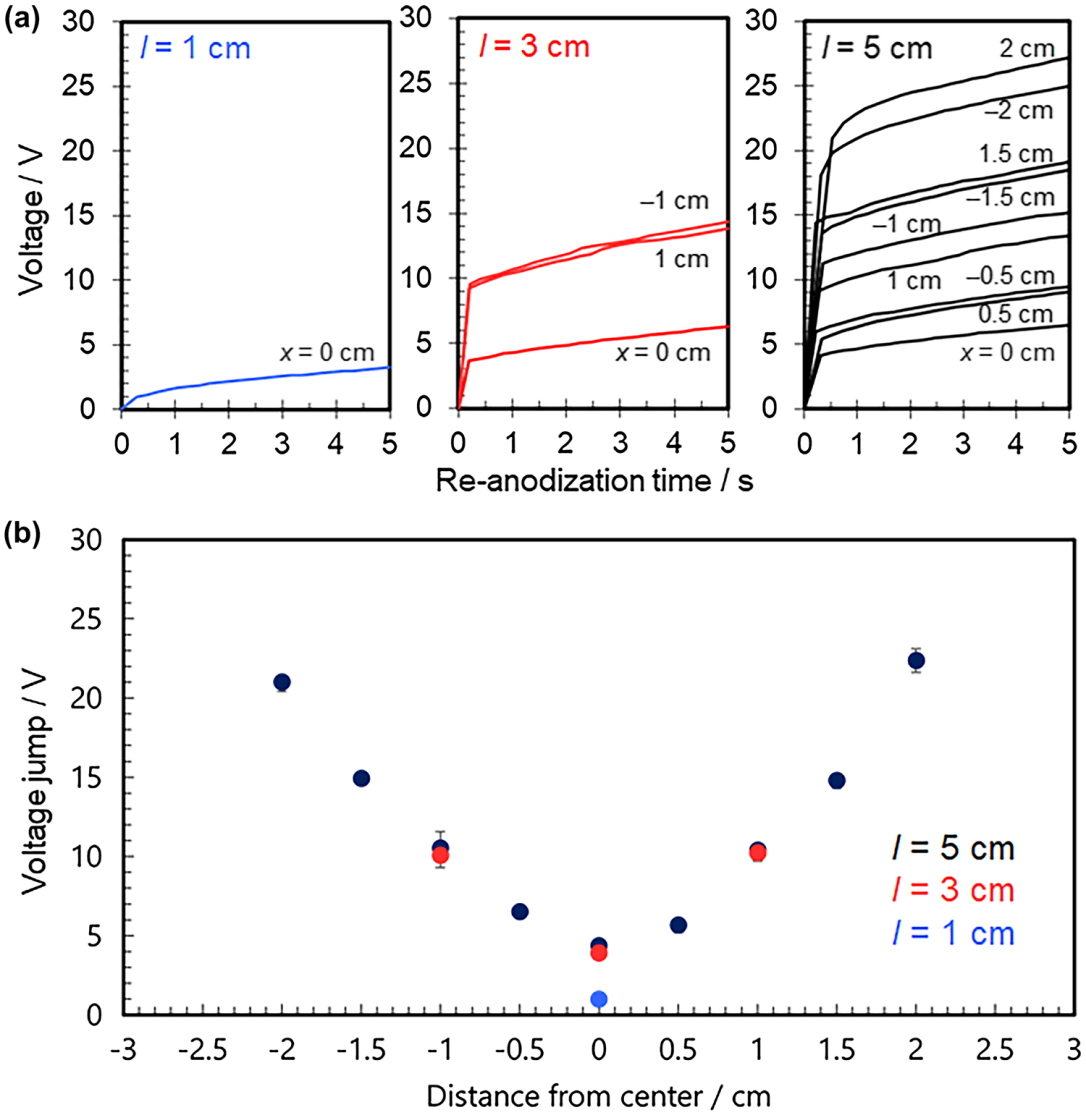
**(a)** Typical voltage–time curves recorded at different locations on the aluminum specimens during re-anodization and **(b)** voltage jump (*V*_j_) plotted as a function of the distance from the center of the BPE. Each reported value for *V*_j_ is the average of three measurements.

To evaluate the thickness distribution of the barrier layers in the porous alumina films formed on each BPE, specimens were cut into 1 × 1 cm^2^ pieces and re-anodized. The *V*_j_ was measured within 8 mm of the center of each square piece. The *V*_j_ value was recorded as the average of the measured values for of each piece. We describe the evaluation principle and re-anodization method in detail in previous papers^20–23^. *V*_j_ from the voltage–time curve recorded during re-anodization should depend on the electrical resistance of the aluminum BPE, which is determined by the thickness of the barrier layer in the porous alumina film. Figure 3a show the initial stage of the voltage–time curves for re-anodization of each specimen. Figure 3b plots *V*j for each region of BPEs with different lengths of *l* = 1–5 cm and a fixed width of *w* = 1 cm. *V*_j_ of a BPE with *l* = 5 cm and *w* = 1 cm, which we reported previously^20^, is also shown for comparison.

The BPE with *l* = 3 cm had *V*_j_ ~ 10 V at both ends (*x* =  ± 1 cm) and ~ 5 V at the center (*x* = 0). This was consistent with the expectation that the interfacial potential difference is highest at the edge. The potential gradient from the center to the edges of the BPE surface was nearly linear. The electrical resistance had a symmetrical distribution centered at *x* = 0 cm, which agreed well with the previous results for the BPE with *l* = 5 cm^20^. The slopes indicated that the electrical resistance of the barrier layer increased in proportion with the applied potential difference on the BPE surface. If the areas of the anodic and cathodic reactions are equal in size, no *V*_j_ should be detected at the center of the BPE. However, electrical resistance was detected there, so the interfacial potential difference was not zero. This may be attributed to an increase in the anodic driving force beyond the center of the BPE during AC bipolar anodization.

The BPE with *l* = 1 cm (i.e., at the center of the square) had *V*_j_ ~ 1.4 V, which is lower than the values for BPE with *l* = 3 and 5 cm even at the same position (x = 0). Thus, the redox reaction was clearly affected by the BPE length. The difference between potentials at the ends of a BPE, ∆*V*_BPE_, was estimated by considering the potential difference *E*^1^:

3 $$\Delta V_{\text{BPE}}=E\times \frac{l}{L}$$

Therefore, BPE with a relatively long *l* should have a large ∆*V*_BPE_, and the degree of polarization should increase. With a DC field, the field direction is simple and fixed, so ∆*V*_BPE_ may be estimated by the length of the BPE alone based on Eq. (3). For an AC field, however, ion migration (both anions and cations) in the electrolyte and electron transfer in the BPE are more complicated because the polarity of driving electrodes (i.e., the field direction) switches periodically. In addition, because the redox reaction occurs on the left and right sides of the BPE at the same time, the reaction at the center may be completely suppressed for a BPE with a relatively short *l*. In other words, the BPE with *l* = 1 cm was expected to have the lowest degree of polarization among the specimens considered in this study.

### Simultaneous processing of multiple aluminum BPEs

**Figure 4:**
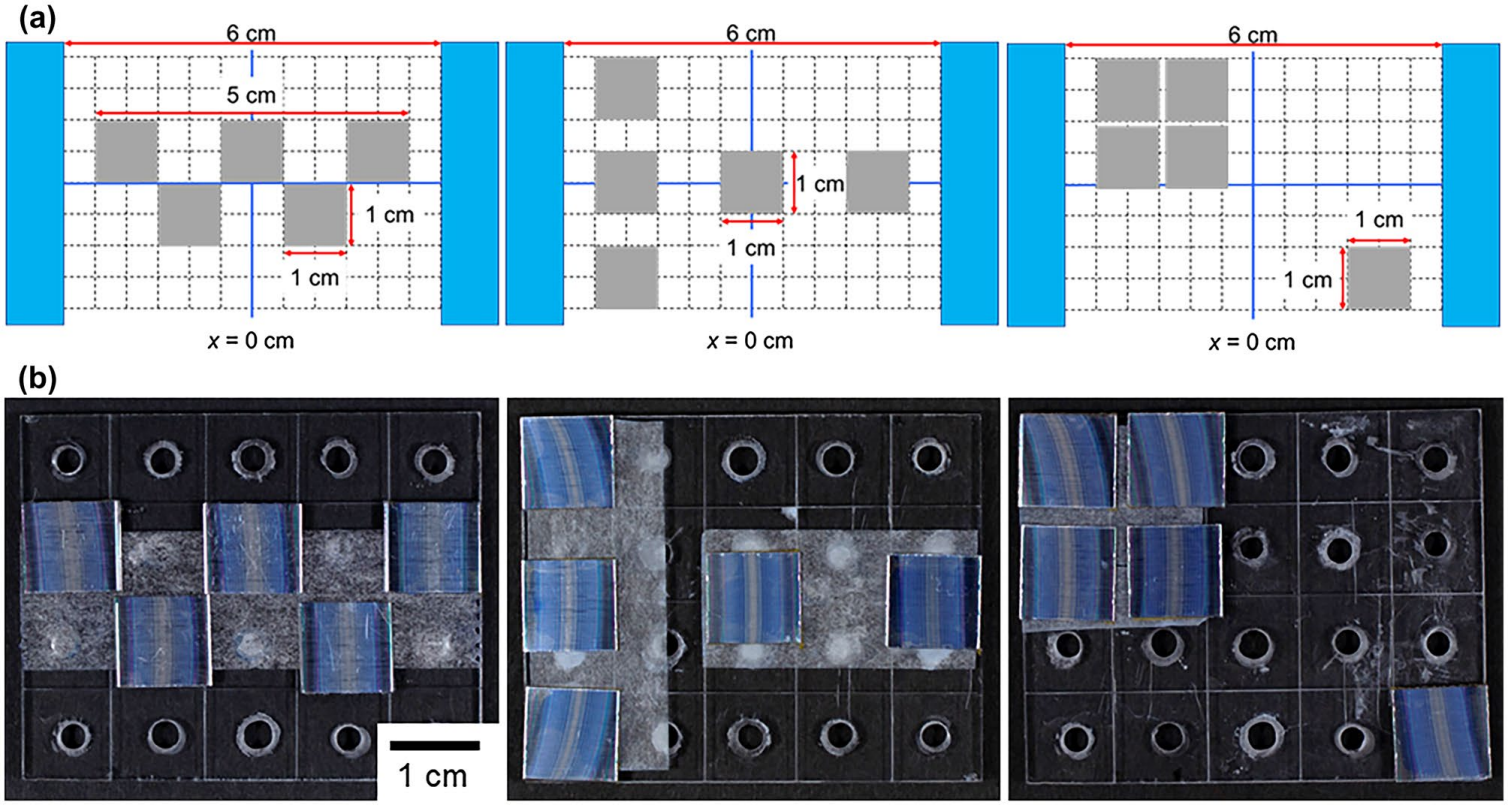
**(a)** Top-view schematic for bipolar anodization of multiple BPEs and **(b)** digital photographs of aluminum BPEs recorded after AC bipolar anodization in 10 mmol dm^−3^ oxalic acid at 20 °C for 60 min at 60 V and subsequent dyeing. The actual BPE positions correspond to those shown in **(a)**.

To clarify the points of note when processing multiple BPEs at the same time, the position of each BPE between the driving electrodes was changed as shown in Fig. 4a. Specific interference fringes were observed to evaluate the equipotential surface. Figure 4 summarizes the position of each BPE and their surface appearances after AC bipolar anodization and dyeing. For all BPE configurations, specific interference fringes parallel to the surfaces of the driving electrodes were observed. The coloration was almost the same as for the BPE of the same size (1 × 1 cm^2^) and treated alone, as shown in Fig. 2. The reproducibility of the experiment processing multiple BPEs was confirmed as shown in Fig. S3. All BPEs had *V*_j_ values of less than 2 V, which is close to the result shown in Fig. 3 (~ 1.4 V).

## Discussion

**Figure 5:**
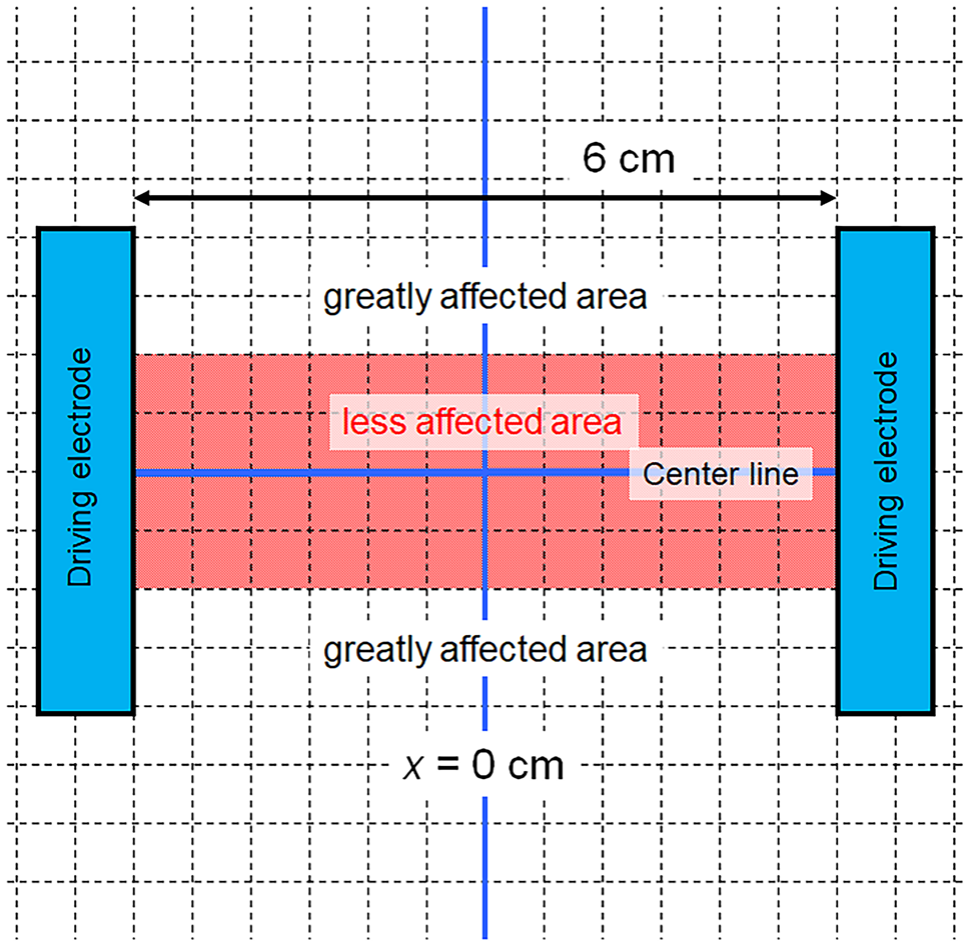
Schematic of the recommended area (in red) to place BPEs in for bipolar anodization. The grid is drawn with a spacing of 5 mm.

For BPEs far from line connecting the centers of the driving electrodes, the interference fringes showed obvious bending, as shown in Fig. 4b. The degree of bending strongly depended on the BPE position between the driving electrodes. This was clearly influenced by the potential lines (electric field lines) generated between the driving electrodes and represented the distribution of the equipotential surface. Although the *V*_j_ values of each BPE were almost the same because measurements were taken at the center of the BPE, the interference fringes were obviously bent by the AC electric field itself. In other words, the BPE position in a non-uniform electric field is an important factor for controlling the uniformity of a redox reaction. Because positions far from the center are greatly affected by the curvature of the potential lines, BPEs should be placed at the center (i.e., less affected area), as shown in Fig. 5

Although it was difficult to measure the equipotential surfaces on the BPE owing to wireless operation, their distribution (i.e., the electric field line pattern) could be simply confirmed visually by the interference fringes. Because the electric field is always perpendicular to the equipotential surface, two equipotential surfaces never intersect. The interference fringes in this study were generally parallel and did not intersect. The edge of a driving electrode was considered to act like a point charge, which generated equipotential surfaces like concentric spherical shells around it. As a result, for BPEs far from the center, the interference fringes showed remarkable bending. The bending of the interference fringes was observed even for wide BPEs (e.g., *w* = 2 and 3 cm), as shown in Fig. 2. These results are important findings for precise control of electrically coupled faradaic reactions when processing not only single but also multiple BPEs according to wireless operations.

## Conclusions

In this study, we investigated the influences of the length and width of rectangular aluminum BPEs on reactions in an AC electric field. The structures of porous alumina films formed on the BPEs were evaluated by dyeing and re-anodization. Specific interference fringes were observed on all anodized specimens before dyeing regardless of the BPE size. The interference fringes were nearly parallel to the surfaces of the driving electrodes and showed linear symmetry on each BPE. The equipotential surface was visualized by observing specific interference fringes on the BPEs. The results show that both the BPE size and position have significant effects on the formation of uniform films and provide useful insights for the simultaneous processing of multiple BPEs. Accordingly, BPEs should be placed at the center of the cell.

With regard to wireless operation, the principles of bipolar electrochemistry and conventional dielectrophoresis are similar, but the configuration of the object with respect to the driving electrodes is important. Our findings provide significant and novel insights into material preparation, sensing, and screening applications based on bipolar electrochemistry. Our hope is that they will inspire new developments in micro-/nano-fabrication and related research.

## Supplementary Information

Supplementary Figures.

## Data Availability

Correspondence and requests for materials should be addressed to H.A.
