# Supplementary material for: Effects of size and position of an unconnected aluminum electrode on bipolar anodization in an AC electric field
Source: Sci Rep. 2021 Nov 18;11:22496. doi: 10.1038/s41598-021-01633-4 (PMC8602422; doi:10.1038/s41598-021-01633-4)
Supplement: Supplementary file 1 — Supplementary Figures. [file 41598_2021_1633_MOESM1_ESM.pdf]

Supplementary Information

for

Effects of size and position of an unconnected aluminum electrode on bipolar anodization in an AC electric field

Ryo Takeuchi, Hidetaka Asoh*

Department of Applied Chemistry, Kogakuin University, 2665-1 Nakano, Hachioji, Tokyo 192-0015, Japan

*Corresponding author. E-mail address: asoh@cc.kogakuin.ac.jp (H. Asoh).

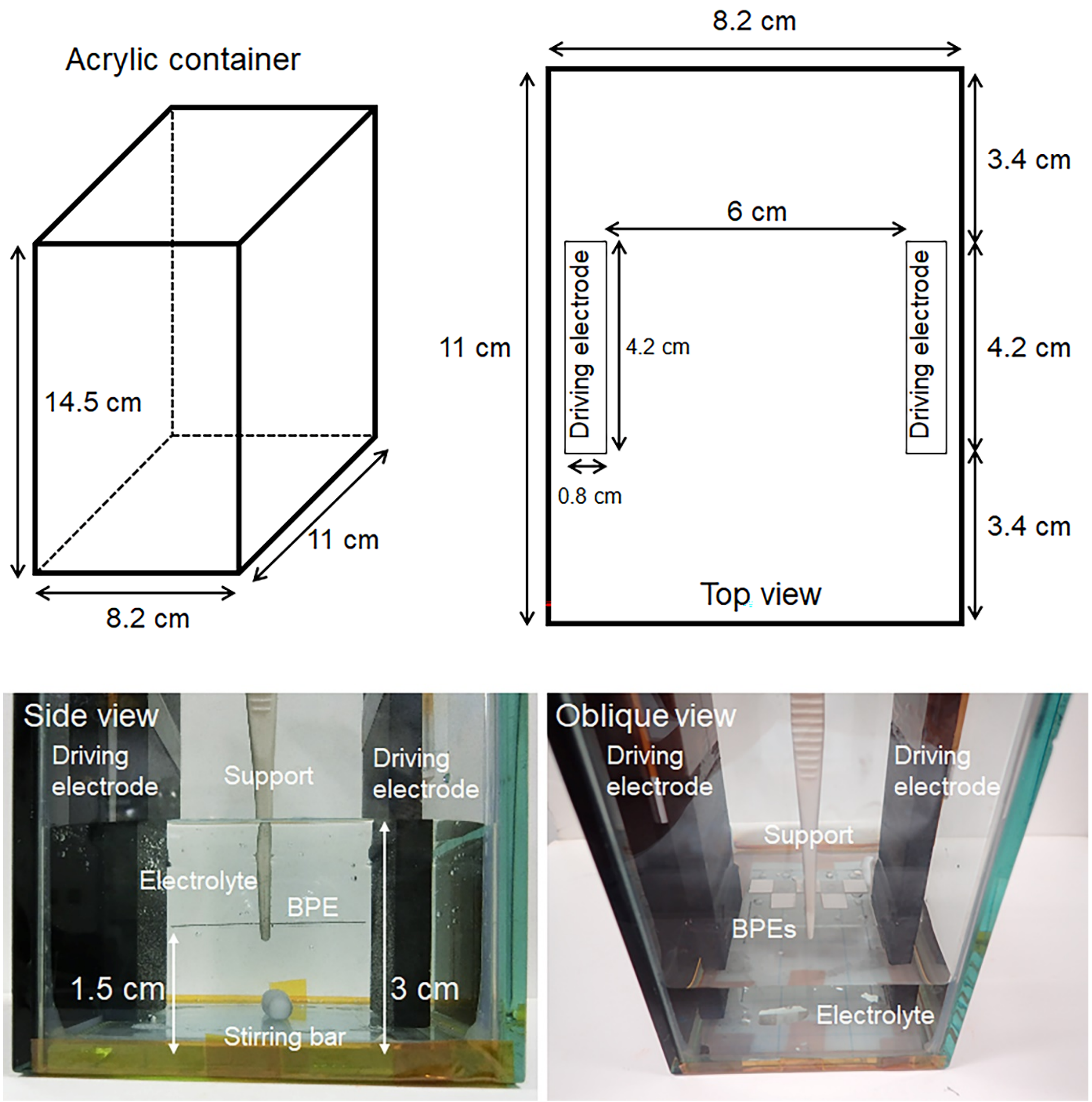

Figure S1. The experimental setup for bipolar anodization. The distance between the carbon driving electrodes was 6 cm. The aluminum BPEs were positioned horizontally at the center of the cell and surrounded by the electrolyte. One side of each BPE was exposed to the electrolyte.

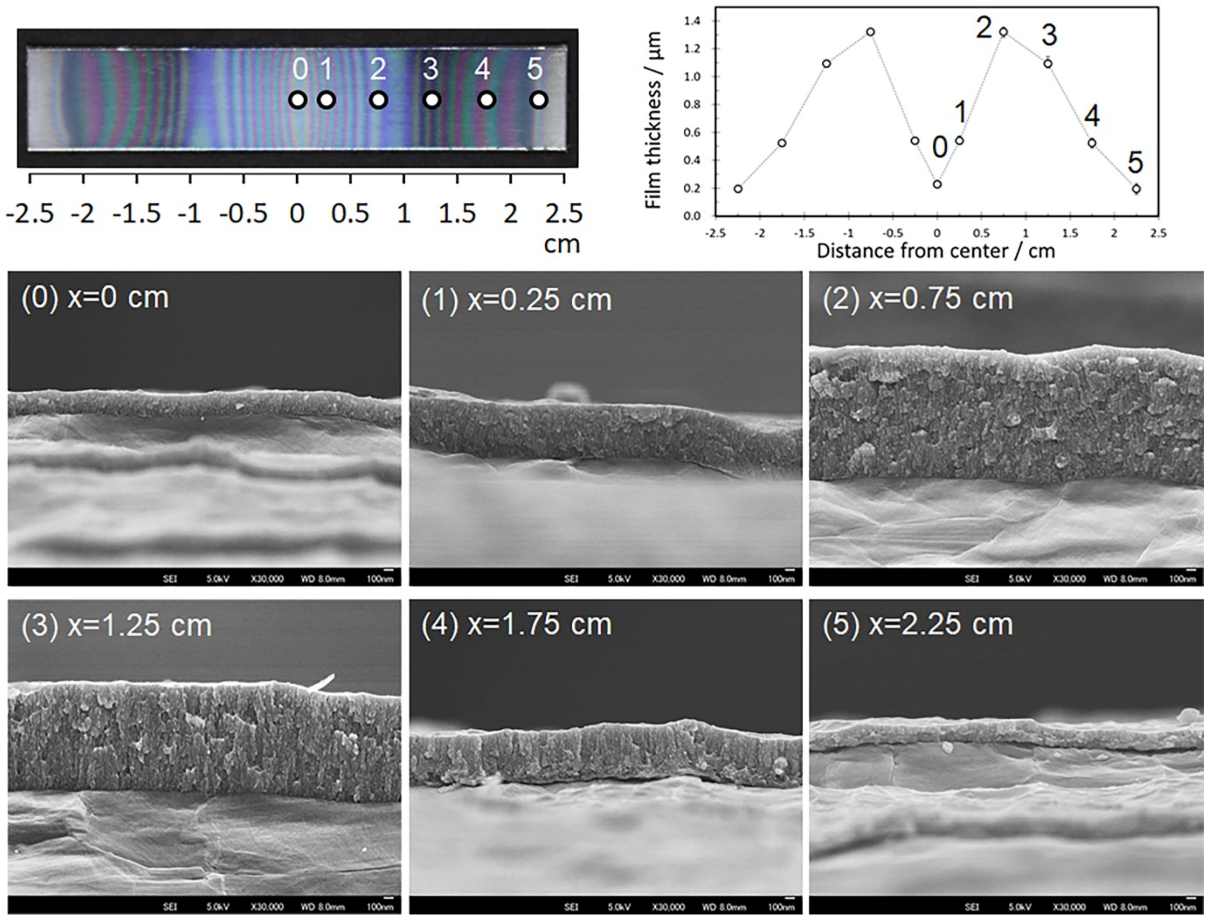

Figure S2. Cross-sectional SEM images of an alumina film at various locations on a BPE with a length of l = 5 cm and width of w = 1 cm, where x denotes the distance from the center of the BPE. AC electrolysis was performed in 10 mmol dm^{-3} oxalic acid at 20 °C and 60 V for 60 min at 150 Hz. The BPE was anodized symmetrically, so data from one half of the BPE were representative.

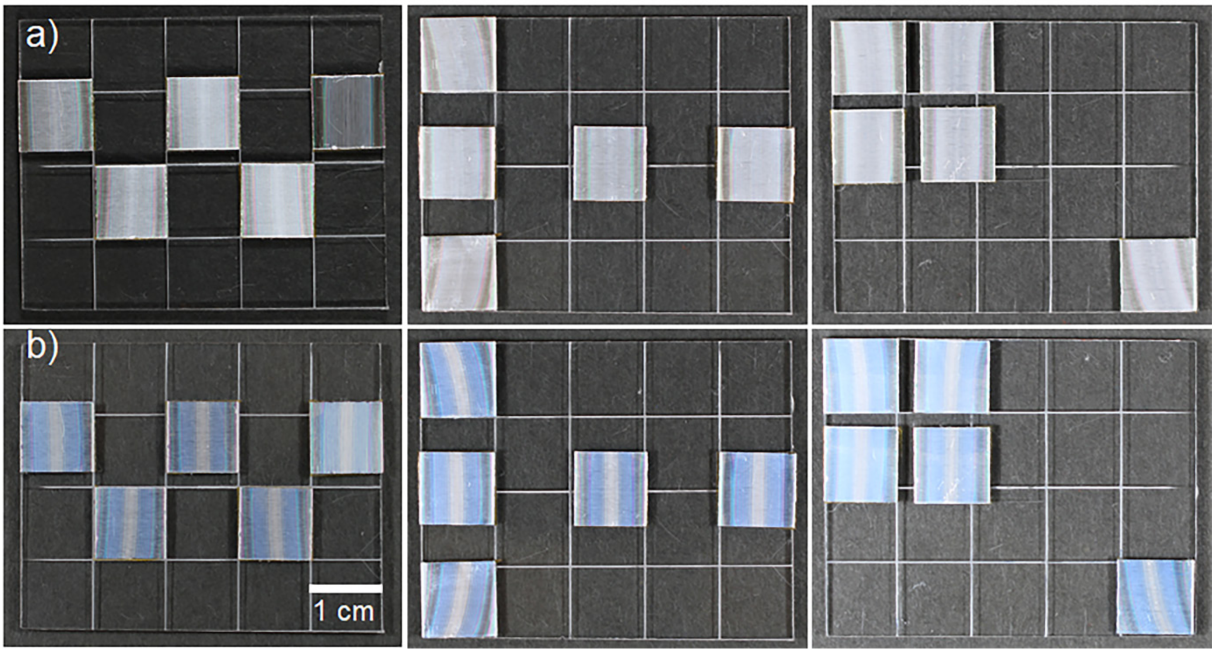

Figure S3. Reproducibility experiment. Digital photographs of aluminum BPEs recorded (a) after AC bipolar anodization and (b) after the subsequent dyeing. AC electrolysis conditions were the same as those in Fig. 4. Here, the BPEs were placed on an insulating plate with no holes and fixed by double-sided adhesive tape.
